# Characteristics of Vitamin D Concentration in Elite Israeli Olympic Athletes

**DOI:** 10.3390/nu16162627

**Published:** 2024-08-09

**Authors:** Ori Abulafia, Elya Ashkenazi, Yoram Epstein, Alon Eliakim, Dan Nemet

**Affiliations:** 1 The Elite Sport Department of Israel, Wingate Institute, Netanya 4290200, Israel; 2 School of Public Health, Faculty of Medical and Health Sciences, Tel Aviv University, Tel Aviv 69978, Israel; 3 Department of Pediatrics, Meir Medical Center, Kfar-Saba 4428164, Israel; 4 School of Medicine, Faculty of Medical and Health Sciences, Tel Aviv University, Tel Aviv 69978, Israel; 5 Faculty of Health Sciences, Department of Sport Therapy, Ono Academic College, Kiryat Ono 3133801, Israel; 6 The Medical Department, Olympic Committee of Israel, Tel Aviv 69706, Israel

**Keywords:** vitamin D, Olympic sports, seasonality

## Abstract

Background: The prevalence of vitamin D deficiency has been a growing concern in recent years. Vitamin D is important in many of the body’s physiological systems, such as the musculoskeletal, cardiovascular and immune functions. A deficiency of vitamin D in athletes may negatively impact both muscle functions and recovery and, thus, affect performance and increase the risk of injury. Many studies assessed the prevalence of vitamin D deficiency in athletes; however, as of today, there are no official recommendations/protocols for screening vitamin D levels in athletes, and only a few studies were performed in male and female elite athletes (i.e., Olympic level), in different sport disciplines. Method: We investigated the prevalence of vitamin D deficiency among athletes entering the Israeli Olympic team. A total of 761 samples of Vitamin D(OH)25 from 334 athletes were analyzed. For this analysis, we used the first test the athlete had performed when joining the Olympic team. The prevalence of vitamin D deficiency (<50 nmol/L, as defined by the Endocrine Society Committee) was investigated according to gender, types of sports and outdoor vs. indoor sports through the different seasons of the Israeli Olympic team athletes. Result: Twenty-five athletes (7.5%) were diagnosed with vitamin D deficiency. One hundred and thirty-one athletes (39.2%) had insufficient levels of vitamin D (50–75 nmol/L). The highest incidence of vitamin D deficiency was found amongst gymnastics and combat sport athletes. A significant difference was also found in vitamin D concentration between seasons. Vitamin D average concentration in the winter was 74.1 nmol/L compared to 86.4 nmol/L in the Summer (*p* < 0.0005). Conclusions: Due to the importance of vitamin D to athletic performance and the high prevalence of deficiency and insufficiency, we suggest careful and frequent monitoring of groups at risk, including elite athletes, especially in susceptible sports and during the winter. Future studies are necessary to investigate the effectiveness of Vitamin D supplementation in athletes with low baseline vitamin D levels.

## 1. Introduction

Vitamin D plays an important role in multiple physiological systems and processes, such as the immune system, protein synthesis, musculoskeletal system, cardiovascular function and inflammatory response [1]. It is suggested that vitamin D may improve athletic performance [2], especially aerobic endurance, anaerobic power and strength in elite athletes [3]. Vitamin D is synthesized in the skin, with daily sunlight exposure of 15–30 min, or absorbed from certain food types, such as fatty fish, egg yolk and dairy products. An individual’s vitamin D status is defined by the serum 25(OH) D concentration. The Endocrine Society Committee (ESC) has defined a concentration of >30 ng/mL (>75 nmol/L) as sufficient, 21–29 ng/mL (52.5–72.5 nmol/L) as insufficient and <20 ng/mL (50 nmol/L) as deficient [4].

Vitamin D is known for its role in calcium homeostasis for optimal skeletal health [5]. When levels of vitamin D decrease below normal limits, Parathyroid hormone (PTH) increases bone resorption to meet the body’s demands for calcium. This means that low levels of vitamin D can lead to secondary hyperparathyroidism and an increase in bone turnover and may cause bone and muscle injury, which are common in athletes [1]. Research to date advocates that certain athletes may be at risk for low vitamin D concentration, which may increase their risk for injuries and suboptimal musculoskeletal function [6].

When comparing the prevalence of vitamin D deficiency in athletes to the general population, data are controversial; some studies report a high prevalence of vitamin D deficiency in athletes [7], while others, in various groups of athletes, suggest that these levels are comparable to the general population [1]. More recent publications show levels that may depend on the type of sport, weather seasons, indoor vs. outdoor sports and even pre- and mid-COVID-19 quarantine period occurrence [8,9]. Ip et al. [4] suggested that vitamin D inadequacy is more prevalent in power sports than in endurance sports. Constantini et al. [10] demonstrated that vitamin D deficiency was more prevalent in dancers and basketball players than in swimmers. Jastrzębska et al. [9] confirmed significant changes in 25(OH)D, Ca, P and PTH concentrations during the annual training season. The maximal values of 25(OH)D concentration were reported at the end of summer. Recently, studies indicated that home isolation during the COVID-19 pandemic in the spring contributed to the persistence of low 25(OH)D concentration in athletes [9]. Saita [8] compared pre- and mid-COVID-19 period vitamin D concentration in a professional Japanese football team and demonstrated that during the spring of 2018, only 3/24 players (13%) were vitamin D insufficient (<30 ng/mL); in contrast, all players (23/23) were vitamin D insufficient in the spring of 2020. This clearly indicates that the stay-at-home orders and restriction of outdoor training resulted in a reduction in vitamin D levels in the athletes. On the other hand, other research failed to demonstrate differences in vitamin D levels between different seasons and indoor or outdoor sports activities [11]. Interestingly, data regarding Olympic-level athletes is limited.

Thus, the aim of the present study was to evaluate the prevalence of Vitamin D deficiency and insufficiency in elite athletes entering the Israeli Olympic team. We hypothesized that vitamin D concentration will be higher in males compared to females, as well as in specific sports environments (e.g., outdoor) and types (endurance).

## 2. Materials and Methods

### 2.1. Participants

Our study population included male and female elite athletes from The Israeli Olympic team between the years 2016 and 2024 (two Olympic periods). Blood test was taken as part of routine examination of the Israeli Olympic team members. The total number of Vitamin D3 25(HO)D serum samples was 761. For the current study, we selected the *first test* of each athlete upon entering the Olympic team and reported overall 334 baseline tests (148 females, aged 13 to 39 years). The study was approved by the Meir Medical Center Helsinki Committee (MMC 0056-19, approved 18 April 2019).

The Israeli National Olympic team includes athletes ranked in the top 16 in European/World Championship or equal international competitions at senior and junior levels. Exclusion criteria included any chronic disease that may affect the blood test results. This study does not include blood samples containing less than 40 biomarker results (non-screening tests) or disease-related tests (blood samples contain unique biomarkers) to avoid tests ordered due to specific clinical reasons. In addition, any blood samples collected on dates other than the predetermined screening test dates were excluded from the study for reasons similar to those discussed above.

### 2.2. Blood Sampling Collection and Analysis

Blood samples were collected by the medical team of the Ribstein Center for Sports Medicine Sciences and Research at the Wingate Institute early in the morning, following 12 h of fasting (with water allowed ad libitum) and at least 12 h from the last workout. After blood sample collection, all tubes were stored in temporary refrigerators and transported immediately to the Meir Medical Center (MMC) labs for analysis. The transport was performed in an insulated cool box under the Ministry of Health’s guidelines.

Blood samples were analyzed at the MMC labs within 24 h after collection. The system used for analyzing the samples is AU5800 (Beckman Coulter Inc., Brea, CA, USA). Vitamin D was analyzed using the LIAISON^®^ 25 OH Vitamin D total Assay, a fully automated immunoassay for the direct measurement of 25 OH vitamin D total levels (DiaSorin, Saluggia, Italy) following the standard biochemical protocol [12]. Samples were analyzed in duplicates with a %CV of <3%. The results were then uploaded to a private cloud safe and stored in an athlete management system, “Smartabase” (Fusion Sport Pty Ltd., Brisbane, Australia), as approved by Clalit Health Services (Tel Aviv, Israel) and the Israeli Ministry of Health.

### 2.3. Type of Sport

In order to compare the different types of sports, we divided the athletes into several categories in the following order:

I. *Anaerobic*: powerlifting and anaerobic track and field.
II. *Endurance*: triathlon, cycling, long-distance running.
III. Endurance strength: gymnastic artistic, climbing, kayaking, rowing.
IV. Fencing: fencing.
V. *Combat*: judo, taekwondo, wrestling, boxing.
VI. Rhythmic gymnastics.
VII. Sailing: sailing, surfing.
VIII. Swimming: swimming.
IX. Synchronized swimming: synchronized swimming.

*Other*: alpine skiing, skateboarding, figure skating, short-track speed skating, basketball, handball, badminton, beach volleyball, tennis, breakdance, modern pentathlon, soccer, archery, baseball, golf, shooting, table tennis.

### 2.4. Data Analysis

Sport types were grouped based on similar physiological characteristics. Athletes not falling into any predefined category were labeled as “Other”. Sports types with less than 10 athletes are also labeled as other. The original sport types, alongside their assigned groups, are presented below.

For each blood marker, the following statistical measures were computed for both genders: mean, standard deviation (STD), percentage of high abnormalities, percentage of low abnormalities and total percentage of abnormalities. Deficiency percentage was measured for vitamin D only. To test the mean difference between genders for each marker, and the difference between outdoor and indoor sport types within each gender for each marker, independent t-tests or Mann–Whitney U tests were used, depending on the assumptions. The effect size was measured by Cohen’s d to complement *p*-values when statistical significance was observed. Linear regression was utilized to assess the influence of gender, sport type, age, season of the year and indoor/outdoor conditions on each blood marker. In cases where the assumptions of linear regression were not met, robust regression techniques were employed to ensure the validity of the analysis. For blood markers exhibiting more than ±8% abnormal results, logistic regression was performed using the same set of variables to explore potential predictors of abnormalities. Only sport types that were extremely lower in the descriptive were chosen for the regression. These sport types were compared to all other athletes in the data to be more stringent than comparing all sport types to each other. Statistical significance is considered as *p* ≤ 0.05. All data were analyzed through Python 3.7 (Python Software Foundation, www.python.org, accessed on 19 July 2024, Wilmington, DE, USA).

## 3. Results

The baseline albumin, calcium and phosphate concentrations (148 females, 186 males) are presented in Table 1. Urea and creatinine levels were within normal range in both females (33 ± 8 mg/dL, 0.9 ± 0.1 mg/dL) and males (39 ± 9 mg/dL, 1.1 ± 0.2 mg/dL), indicating proper hydration [13]. We found no abnormal levels of albumin and only a few cases of abnormal calcium and phosphate concentrations.

Overall, 156 athletes had abnormally low vitamin D serum concentration (47%). The results of the baseline vitamin D status are presented in Table 2.

### 3.1. Female vs. Male Comparison

Significantly higher vitamin D, Ca and albumin levels were found in male athletes (Table 3).

### 3.2. Sport Categories

Vitamin D concentration and abnormal results by sport categories (with males and females separately listed) are presented in Table 4.

### 3.3. Indoor vs. Outdoor

Vitamin D concentrations in indoor and outdoor sports are presented in Table 5. A significant difference was found both in females and males in vitamin D levels between indoor and outdoor sports. This difference is mainly driven by combat sports and artistic gymnastics.

### 3.4. Season Comparison

The winter season was defined as between November and March. The summer season was defined as between April and October. We used the data from athletes who joined the Olympic team in the summer and compared them to those who joined in the winter.

The average winter vitamin D level was 74.08 nmol/L compared to the summer average level of 86.37 nmol/L, demonstrating significantly higher levels in the summer season with a *p* value ≤ 0.0005.

### 3.5. Multiple Regression Analysis

In a multiple regression analysis (Table 6), summer was found to be protective against vitamin D deficiency, while combat sports and rhythmic gymnastics were found to significantly increase the risk of vitamin D deficiency and insufficiency.

## 4. Discussion

Our study demonstrated a high overall prevalence of inadequate vitamin D levels in Israeli elite Olympic athletes entering the Olympic team. Vitamin D deficiency was found in 7.5% (*n* = 25) of athletes, and insufficiency was found in 132 athletes (39.5%). We were able to detect sports in which athletes were more prone to having low vitamin D concentrations.

While it may appear that elite athletes possess adequate levels of vitamin D since many of them are monitored by dietitians and are exposed to sunlight, recent research challenges this assumption. Over the past decade, several studies have investigated vitamin D levels among different groups of athletes, such as runners, basketball players, jockeys, gymnasts and dancers, revealing that Vitamin D concentrations in athletes are similar or even lower compared to the general population [1]. In a recent study on the Norwegian Olympic team [14], vitamin D was found to be the most common blood sample abnormality affecting one-third of the athletes. Our study supports the notion that Vitamin D is insufficient in a large number of elite athletes, thus mandating its frequent evaluation and possible supplementation if needed.

In contrast to our first hypothesis, we were unable to find a significant gender difference in vitamin D concentrations. This is consistent with the systemic review and meta-analysis by Tilda Harju et al. [15], gathering eight studies that concluded that no vitamin D gender difference exists. Several studies attempted to evaluate levels of vitamin D deficiency in different sports disciplines; however, most observed the same levels of insufficiency between the different sports types [1]. In contrast, it was suggested that vitamin D inadequacy is more prevalent in power sports (65.4%) than endurance sports (32.9%) [4]. Other studies demonstrated a higher prevalence of vitamin D deficiency in dancers and basketball players [8,10] but did not compare them with other sports types. In order to better understand the risk factors for vitamin D deficiency in relation to the sports disciplines, we divided our athletes into several groups: anaerobic, endurance, endurance strength, fencing, combat, rhythmic gymnastics, sailing, swimming, synchronized swimming and other.

Comparing the above groups, we found that vitamin D deficiency and insufficiency were more prevalent among females engaged in combat sports (28%, 48%), males engaged in combat sports (11%, 44%) and females engaged in rhythmic gymnastics (14%, 64%). While this difference may be explained by the fact that rhythmic gymnastics and combat sports are performed indoors, we did not find similar results in other indoor sports, such as fencing. Vitamin D deficiency in rhythmic gymnastics and combat sports may be especially important due to the possible role of vitamin D in force and power production. Interestingly, low vitamin D concentration was also found in endurance male athletes. The low levels of vitamin D among male endurance runners are probably due to the fact that many Israeli elite runners are of Ethiopian descent, and dark-skinned athletes have a significantly higher risk of vitamin D deficiency [6]. Previous studies are controversial in demonstrating if there is a significant difference in the vitamin D deficiency prevalence between indoor and outdoor sports. Some studies fail to show a significant difference between indoor and outdoor [7], while others demonstrated significant differences [8]. Our study found a significant difference between the average vitamin D concentration between some indoor and outdoor sports disciplines. Yet, as a group, we were unable to detect a difference between indoor and outdoor sports.

While Rybchyn et al. suggested that 25(OH)D cycles in and out of muscle cells and appears to be upregulated in the winter, protecting those with higher muscle mass [16], most studies agree that 25(OH)D production and concentration is higher in the summer [10,11]. Therefore, it is not surprising that our study also demonstrated higher average levels of vitamin D in the summer compared to the winter season. Israel is a sunny country; even in the winter, we can only assume that winter clothing affects solar ultraviolet exposure and, thus, leads to lower winter levels [17]. This variation in vitamin D concentration throughout the year mandates repeated evaluation of vitamin D during different seasons of the training and competitive year and perhaps even specific periods of vitamin D supplementation if indicated.

Bone mass accrual occurs mainly during the second and third decades of life, with many athletes in this age group. The long-term effect of vitamin D deficiency or insufficiency in this important period of bone growth on the future risk of bone abnormalities and other diseases [18] has yet to be determined.

While our study included a relatively large number of elite athletes (334 athletes), its main limitation is the relatively small number of athletes in each sports category. To overcome this, we gathered similar sports into groups, and as a result, most of the categories were sufficient in numbers. Vitamin D screening evaluation is relatively new, and we are continuing to collect data and plan a future study that will include additional athletes in the different sports events. It is important to note that our study population is a unique group of Olympic-level athletes. In addition, the study examined vitamin D concentration only when the athletes entered the Olympic team. Continued measurements throughout the training and competitive season and the response to vitamin D supplementation could add important information to this unique group of elite athletes [3].

## 5. Conclusions

In summary, our study suggests that vitamin D deficiency and insufficiency are very common in Olympic Olympic-level athletes entering the Olympic team. This is more prevalent among gymnastics and combat sports, and in the winter compared to the summer season. Due to the importance of vitamin D to athletic performance, we suggest careful monitoring for at at-risk groups and vitamin D supplementation in those susceptible groups of athletes. It is important to note that these are baseline levels at the entrance of the athletes to the Olympic team. Many athletes with low baseline levels are being supplemented now with vitamin D. A future study is necessary to investigate the supplementation effect on the athletes throughout the competitive year.

## Figures and Tables

**Table 1:** Baseline albumin, calcium and phosphate levels (*n* = 334, 148 females).

Biomarker	Gender	High (n)	Low (n)	Normal (n)	High (%)	Low (%)	Abnormal (%)	Mean
Albumin 3.5–5.5 g/dL	Female	0	0	148	0.0	0.0	0.0	4.3 ± 0.3
	Male	0	0	186	0.0	0.0	0.0	4.5 ± 0.2
Calcium 8.5–10.5 mg/dL	Female	0	0	148	0.0	0.0	0.0	9.6± 0.3
	Male	4	0	182	2.2	0.0	2.2	9.8 ± 0.4
Phosphate	Female	4	0	144	2.7	0.0	2.7	4.1 ± 0.5
	Male	8	1	177	4.3	0.5	4.8	4.0 ± 0.5

**Table 2:** Vitamin D status, normal levels, deficiency and insufficiency.

Vitamin D 25OH Status		All	Female	Male
Deficiency <50 nmol/L	n	25	15	10
	%	7.5%	10.1%	5.4%
Insufficiency ≥50 <75 nmol/L	n	131	60	71
	%	39.2%	40.5%	38.2%
Normal ≥75 nmol/L	n	178	73	105
	%	53.3%	49.3%	56.5%
Total (n)		334	148	186

**Table 3:** Vitamin D, Ca, Phos, Alb concentration—comparing all males and females within the Olympic team.

	Female	Male	*p* Value	Effect Size
Vitamin D (nmol/L)	76.9 ± 24.2	84.2 ± 26.0	0.01	0.3
Ca (mg/dL)	9.6 ± 0.3	9.8 ± 0.4	0.00	0.7
Phos (mg/dL)	4.1 ± 0.5	4.0 ± 0.5	0.07	
Alb (gr/dL)	4.3 ± 0.3	4.5 ± 0.2	0.00	0.7

**Table 4:** Vitamin D deficiency prevalence in different sports disciplines.

Sport	Gender	N	Mean (std) nmol/L	Deficiency (n)	Insufficiency (n)	Normal (n)	Insufficiency (%)	Deficiency (%)
Anaerobic ^c^	F	11	92.0 ± 22.4	0	1	10	9%	0%
	M	11	73.0 ± 21.4	2	4	5	36%	18%
Endurance ^a^	F	10	80.4 ± 12.7	0	3	7	30%	0%
	M	36	88.6 ± 31.1	3	10	23	28%	8%
Endurance ^b^ strength	F	5	66.1 ± 27.6	2	1	1	25%	50%
	M	20	79.1 ± 27	1	9	7	53%	6%
Fencing ^b^	F	12	84.0± 28.3	0	6	6	50%	0%
	M	14	77.2 ± 23.5	0	9	5	64%	0%
Combat ^b,*,**^	F	29	66.1 ± 25.7	8	14	7	48%	28%
	M	36	72.6 ± 19	4	16	16	44%	11%
Gymnastics Rhythmic ^b,*,**^	F	28	64.2 ± 16	4	18	6	64%	14%
Sailing ^a^	F	13	84.9 ± 23.7	0	5	8	38%	0%
	M	14	98.1 ± 31.1	0	4	10	29%	0%
Swimming ^b^	F	12	87.7 ± 15.5	0	1	11	8%	0%
	M	33	95.9 ±21.8	0	6	27	18%	0%
Synchronized Swimming ^b^	F	14	88.6 ± 21.8	0	4	10	29%	0%
Other ^c^	F	14	80.1 ± 29.4	1	7	7	47%	7%
	M	22	84.0 ± 21.3	0	13	12	52%	0%

a—Outdoor athletes, b—indoor athletes, c—group contained both outdoor and indoor athletes; * sport type mean significantly different (*p* < 0.05); ** sport type abnormal results % significantly different (*p* < 0.05).

**Table 5:** Vitamin D serum concentration in indoor and outdoor sports, differing between males and females.

	Female				Male			
	In (*n* = 109)	Out (*n* = 39)	*p* Value	Effect Size	In (*n* = 109)	Out (*n* = 77)	*p* Value	Effect Size
Alb (gr/dL)	4.3 ± 0.3	4.3 ± 0.2	0.47		4.5 ± 0.2	4.5 ± 0.3	0.46	
Ca (mg/dL)	9.6 ± 0.3	9.7 ± 0.3	0.05	0.4	9.8 ± 0.4	9.8 ± 0.4	0.99	
Phos (mg/dL)	4.1 ± 0.5	3.9 ± 0.5	0.06		4.1 ± 0.6	3.8 ± 0.5	0.00	0.6
Vit D (nmol/L)	73.9 ± 24.7	85.5 ± 21.1	0.01	0.5	80.9 ± 23.9	88.8 ± 28.1	0.04	0.3

**Table 6:** Multiple regression analysis (McFadden’s R-squared—0.09).

Variable	β Coefficient	OR (95% CI)	*p* Value
Age	0.03	1.03 (0.97, 1.08)	0.37
Gender (male)	−0.04	0.96 (0.59, 1.56)	0.86
Outdoor sport	−0.33	0.72 (0.42, 1.24)	0.23
Season (summer)	−0.80	0.45 (0.28, 0.72)	0.00
Combat sport	0.89	2.44 (1.29, 4.6)	0.01
Rhythmic gymnastics	1.80	6.07 (2.13, 17.31)	0.00

## Data Availability

The data presented in this study are available on request from the corresponding author (because of privacy restrictions).
